# Modeling marine cargo traffic to identify countries in Africa with greatest risk of invasion by *Anopheles stephensi*

**DOI:** 10.1038/s41598-023-27439-0

**Published:** 2023-01-17

**Authors:** Jordan Ahn, Marianne Sinka, Seth Irish, Sarah Zohdy

**Affiliations:** 1 Rollins School of Public Health, Emory University, Atlanta, GA USA; 2 Centers for Disease Control and Prevention, Atlanta, GA USA; 3 Department of Biology, Oxford University, Oxford, UK; 4 Swiss Tropical and Public Health Institute, Allschwil, Switzerland; 5 US President’s Malaria Initiative, Centers for Disease Control and Prevention, Atlanta, GA USA

**Keywords:** Animal migration, Ecological epidemiology, Ecological networks, Invasive species, Malaria

## Abstract

*Anopheles stephensi*, an invasive malaria vector native to South Asia and the Arabian Peninsula, was detected in Djibouti’s seaport, followed by Ethiopia, Sudan, Somalia, and Nigeria. If *An. stephensi* introduction is facilitated through seatrade, similar to other invasive mosquitoes, the identification of at-risk countries are needed to increase surveillance and response efforts. Bilateral maritime trade data is used to (1) identify coastal African countries which were highly connected to select *An. stephensi* endemic countries, (2) develop a prioritization list of countries based on the likelihood of *An. stephensi* introduction through maritime trade index (LASIMTI), and (3) use network analysis of intracontinental maritime trade to determine likely introduction pathways. Sudan and Djibouti were ranked as the top two countries with LASIMTI in 2011, which were the first two coastal African countries where *An. stephensi* was detected. With Djibouti and Sudan included as source populations, 2020 data identify Egypt, Kenya, Mauritius, Tanzania, and Morocco as the top countries with LASIMTI. Network analysis highlight South Africa, Mauritius, Ghana, and Togo. These tools can prioritize efforts for *An. stephensi* surveillance and control in Africa. Surveillance in seaports of identified countries may limit further expansion of *An. stephensi* by serving as an early warning system.

## Introduction

Globalization and the movement of humans and goods has facilitated the introduction of organisms to new locations, and the list of invasive species has grown substantially since the 1980s^1^. From 2006 to 2014, the movement of maritime shipping between socio-economic regions, defined as maritime countries grouped by similar socio-economic factors, increased by 258% with projected growth of maritime movement of 240% to 1209% by 2050^2^. However, invasive species are not limited to organisms like zebra mussels^3^, pine and eucalyptus trees^4,5^, and feral hogs^6^. Invasive species can also and do often include arthropod vectors of disease and microbial agents, posing significant public health threats. A good example is the introduction of *Aedes aegypti*, the yellow fever mosquito, through the movement of ships in the nineteenth century to the Americas^7^. In the twentieth century, further movement of cargo ships, in particular those carrying used tires, facilitated the spread of *Ae.* spp., including *Ae. albopictus*, a successful invasive species, which has now invaded six continents. The proposed mechanisms facilitating the success of *Aedes* spp. invasion on ships include a few characteristics common to *Ae. aegypti* mosquitoes: the use of artificial containers as larval habitats, the preference for human blood meal, and the ability for eggs to resist desiccation in the absence of water. This drought tolerance has also been used as an explanation for why *Aedes* species can be spread by sea, where other species may require more rapid transportation, such as air travel, for introduced populations to survive long enough to establish in a new location. In the past, species of the *Anopheles* genus of mosquitoes have also been accidentally introduced to non-native countries such as Egypt and Brazil. The successful eradication of *An. arabiensis* in Brazil required a well-coordinated control program following malaria outbreaks^8^.

Unlike *An. arabiensis*, *An. stephensi*, is a unique malaria vector because of its ability to thrive in artificial containers in urban environments. This species is found across South and South-East Asia and the Arabian Peninsula, where it is a primary malaria vector and responsible for both urban and rural malaria transmission. Most malaria vector control efforts are focused on rural habitats, and the ability for malaria vectors to thrive in urban environments may threaten progress made on malaria control and elimination.

In 2012, *An. stephensi* was first detected on the African continent in a livestock quarantine station in a seaport in Djibouti^9^. By 2016, it was then detected in neighboring Ethiopia^10^. By 2018^11^ or 2019^12^, *An. stephensi* was detected near seaports in Sudan and Somalia^13^. It was also most recently detected in Nigeria in 2020^14^. With *An. stephensi* having unique ecological characteristics, and the first detection of the species in seaports, it has been hypothesized that *An. stephensi* introduction was likely facilitated through maritime trade. Further supporting the similarities between *An. stephensi* and *Ae. aegypti* may be the fact that in Ethiopia, a large percentage (40%^15^ or greater^16^) of the habitats where *An. stephensi* larvae were detected, *Ae. aegypti* was also detected. With invasive *An. stephensi* populations now established in these countries, there is a new threat to malaria control on the African continent. Population genetic analyses suggest the potential source of introduction is South Asia^17^.

The invasion of this malaria vector has the potential to significantly impact global malaria control and elimination efforts^18^. For example, in Djibouti, *An. stephensi* has been linked to malaria outbreaks in 2013^9^ and since initial detection in Djibouti, malaria cases have increased 30-fold^19^. Additionally, although it shows a seasonal variability in abundance in Asia, it has been detected year-round through the hot, dry season in Africa^20^. Recent laboratory studies on invasive Djiboutian and Ethiopian *An. stephensi* specimens reveal that as in Asia, these populations are competent vectors for both *Plasmodium vivax* and *Plasmodium falciparum*^20^. Thus, countries may need to expand their malaria testing protocol. Further, field data have shown confirmation of *P. vivax* sporozoites in *An. stephensi* in Ethiopia^21^, and high levels of resistance to nearly all insecticides used in malaria vector control.

Urban centers of sub-Saharan Africa tend to have lower malaria transmission rates than rural areas. However, urbanization in these areas increase breeding habitats and primary vector diversity which will lead to higher risk of transmission^22,23^. A recent habitat suitability modeling study predicted that the further invasion of *An. stephensi* into urban locations on the African continent could put an additional 126 million people at risk of malaria^24^.

To address this global challenge and proactively mitigate the threat of *An. stephensi,* prioritization activities are necessary to identify where this invasive mosquito is likely to be introduced, particularly if movement is facilitated by the movement of cargo through marine shipping. To better understand the invasion of *An. stephensi*, we describe: (1) United Nations Conference on Trade and Development (UNCTAD) maritime trade data from 2011, prior to the detection of *An. stephensi* in Djibouti, and habitat suitability to determine whether historical connectivity identify Djibouti and Sudan as high risk countries for *An. stephensi* introduction, (2) a prioritization list of coastal African countries for immediate surveillance based on 2020 data to allow for early detection, rapid response, and limit further introduction of the vector in Africa, and (3) an interactive network model of intracontinental transport routes in Africa allowing for future prioritization hierarchies for surveillance if/when *An. stephensi* is detected in new locations*.*

## Materials and methods

### Days at sea, habitat suitability index, trade index

Due to the initial detection of *An. stephensi* in the port city of Djibouti City, maritime trade data were examined. We ranked the maritime trade connection between countries with known *An. stephensi* populations (India, Pakistan, Saudi Arabia, and United Arab Emirates) and coastal African countries. Other countries with *An. stephensi* populations such as Iraq, Iran, and Thailand, exhibited lower trade levels and were not included.

We used UNCTAD’s Liner Shipping Bilateral Connectivity Index (LSBCI), an index created from trade data from MDS Transmodal (https://www.mdst.co.uk), to measure the amount of connectivity between each pair of countries. The LSBCI factors in five maritime trade indicators. The first is the number of transshipments, when goods are unloaded and moved to another vessel, to get from country *j* to country *k*. Secondly, LSBCI factors in the number of countries which have direct routes to both countries in the pair (e.g., four countries have direct connections to both country *j* and country *k*). The third indicator is the number of common connections with one transshipment shared between the countries. The level of competition on services that connect the countries, measured by the number of carriers operating on this route, serves as another indicator. Finally, the size of the largest ship on the route with the fewest carriers is considered in calculating LSBCI for a country pair, which can serve as a metric of capacity on sea routes. Each indicator is normalized by subtracting the minimum value from the raw value and dividing by the range. LSBCI is the simple average of the normalized value of these five indicators^25^. The inability to examine specific ports of call or transshipments is a limitation of this dataset. Future examinations of trade and *An. stephensi* may benefit from paid datasets from maritime trade operators.

We took the LSBCI value and divided it by the number of days required to travel by shipping vessel between the closest and largest ports of the countries. This was calculated via *Searoutes* which uses the automatic identification system (AIS) of vessels to track them and calculate average time between ports^26^. The same vessel speed was used in this calculation to maintain uniformity in measuring distance. This compiled index which includes (1) maritime trade degree of connectivity and (2) time between ports (in days) will be referred to as the likelihood of *An. stephensi* introduction through maritime trade index (LASIMTI).

$${\text{LSBCI}}/{\text{Days between countries}} = {\text{LASIMTI}}$$

Additionally, we incorporated Sinka et al.’s Habitat Suitability Index (HSI), which uses, in order of importance, annual mean temperature, population density, seasonal precipitation, surface wetness, vegetation, and other environmental factors to evaluate locations with suitable environments for *An. stephensi* habitation^24^. Using R (https://www.r-project.org/), a data set of countries was ranked by LASIMTI as well as both LASIMTI and the HSI.

The UNCTAD trade data from three years—2011, 2016, and–2020—were chosen. 2011 was selected because it was one year prior to first detection of *An. stephensi* in the Horn of Africa in Djibouti City. In 2016, *An. stephensi* was confirmed in Ethiopia potentially indicating further intracontinental spread or separate introductions. Ethiopia is landlocked and therefore was not included in this study. Finally, maritime trade data from 2020 was evaluated to assess further spread along this pathway. Potentially important to note, the UNCTAD estimates that maritime trade fell by 4.1% in 2020 due to the COVID-19 pandemic. However, they also predict a rebound of 4.8% in 2021^27^.

Maritime trade data from 2020 was used to create a network model of intracontinental African trade between coastal African countries. The connectivity of coastal African nations was examined based on country pairs’ LSBCI. The top three countries, as ranked by LSBCI for each country, were highlighted as links between the nodes. In cases of ties, both countries were included (e.g. Sudan has four country pairs because Egypt, Kenya, and Morocco had the same LSBCI). Another network model was created with a cutoff of 14 days of travel between each node as historical reports show that *An. stephensi* eggs can resist desiccation in soil for up to 14 days^28^ (Supplemental Fig. 1). Edges are weighted by the LSBCI value and nodes are weighted by the number of connected countries. Djibouti and Sudan are differentiated due to their established *An. stephensi* populations. This network model was created with r in RStudio utilizing the igraph and visNetwork packages.

Network centrality is often calculated with eigenvector centrality, which measures the influence of nodes by factoring in the number of connections and the number of connections of its neighbors. PageRank is a variant of eigenvector centrality which considers the direction of edges making it useful for understanding trade^29^. PageRank was used for this network model because of the directed, weighted edges. This rank value determines the centrality of a single node in a network based upon how many connections point towards and away from the node as well as each of its neighbors’ total number of connections. Edge weights and values of other nodes are factored in as well. The PageRank value ultimately is a probability distribution of the nodes in the network. In this network this would essentially be if a single vessel was selected, the probability that it would be found at a given node. PageRank was calculated in RStudio with the igraph package.

### Ethics approval and consent to participate

Not applicable.

## Results

### Maritime index in 2011 prior to detection of An. stephensi in Africa identifies Sudan and Djibouti as highest for risk of introduction

2011 Maritime trade data from UNCTAD point to Sudan and Djibouti as the top two connected countries to the source populations (India, Pakistan, Saudi Arabia, and UAE) when the LASIMTI is summed. The next three countries are Egypt, Kenya, and Tanzania (Table 1, full table: Supplemental Table 1). When HSI is included the top five remain the same (Supplemental Table 2).

**Table 1:** Top 10 Countries of LASIMTI based on 2011 UNCTAD data.

Rank	African Country	Sum of LASIMTI
1	Sudan	0.406
2	Djibouti	0.201
3	Egypt	0.188
4	Kenya	0.081
5	Tanzania	0.073
6	Morocco	0.068
7	Mauritius	0.065
8	South Africa	0.057
9	Comoros	0.055
10	Mozambique	0.054

### Maritime index in 2016 following detection of An. stephensi in Djibouti and Ethiopia highlights Sudan at highest for risk of introduction

2016 UNCTAD maritime trade data shown in Supplemental Table 3 highlight, in order, Sudan, Djibouti, Egypt, Mauritius, and Kenya when ranked by the sum of LASIMTI to the source populations. When this data is ranked first by HSI, the top 5 countries are Sudan, Djibouti, Egypt, Kenya, and Tanzania (Supplemental Table 4).

*Anopheles stephensi* was established in Djibouti in 2012 so after this date, Djibouti can be included as a source population which gives the top five countries as Sudan, Egypt, Mauritius, Kenya, and Tanzania when ranked by their sum of LASIMTI to each source population (Supplemental Table 5). The top five countries when ranked by HSI and then LASIMTI sum are Sudan, Egypt, Kenya, Tanzania, and Morocco when Djibouti is included as a source population (Supplemental Table 6).

### Maritime index in 2020 following detection of An. stephensi in Djibouti, Ethiopia, Somalia and Sudan highlight Kenya, Tanzania, and Mauritius at highest risk of introduction

**Figure 1:**
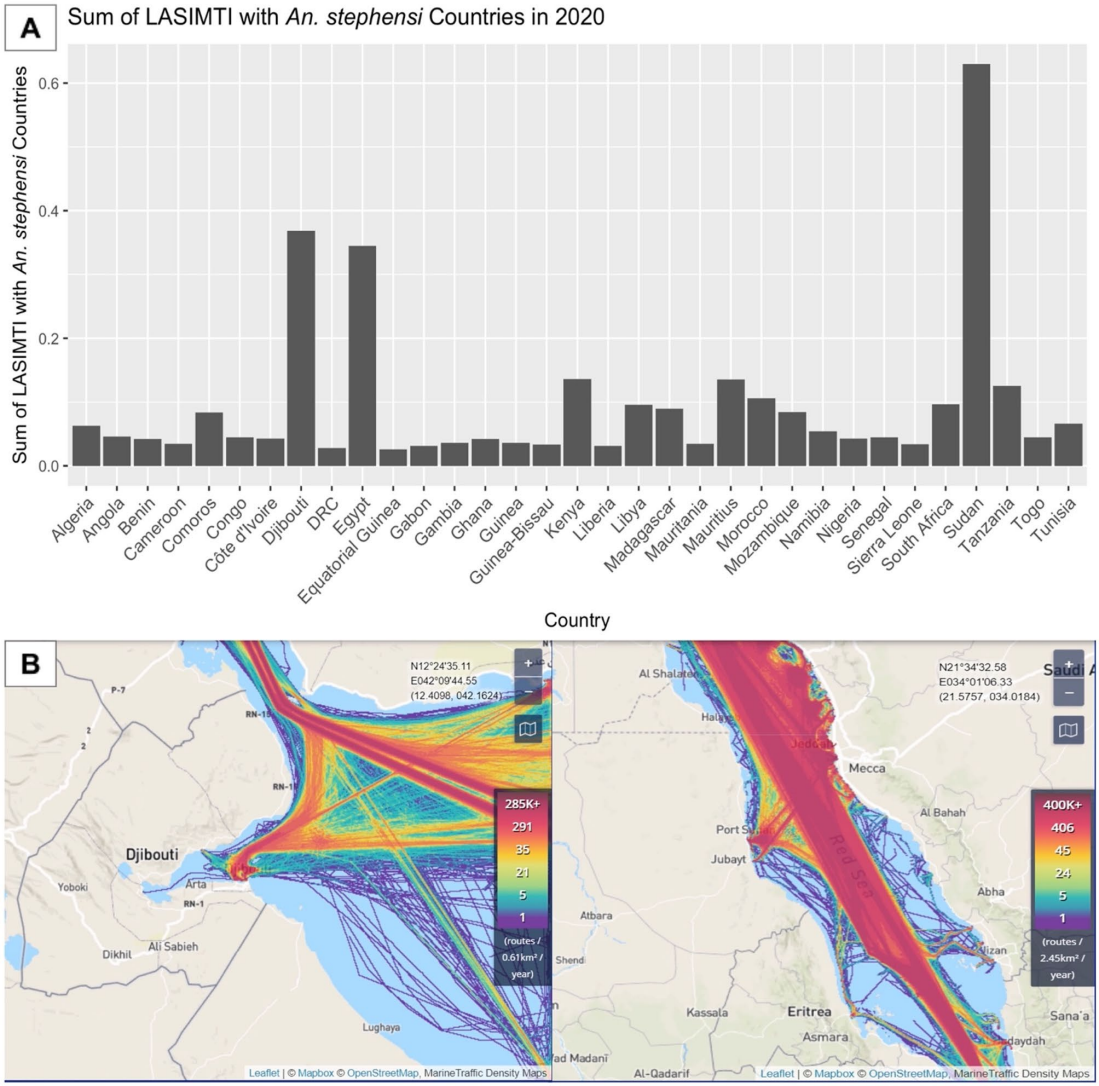
Sum of LASIMTI with *An. Stephensi* countries with 2020 Trade Data. The Likelihood of *An. stephensi* Introduction through Maritime Trade Index (LASIMTI) of coastal African countries in 2020 shows heterogeneity across the continent in maritime movement into ports. (**A**) Relatively high traffic from countries where *An. stephensi* is endemic to Egypt, Djibouti, and Sudan. (**B**) Visualization of the volume of traffic into Djibouti and Sudan in 2019 (modified from marinetraffic.com) shows that a few ports in these two countries accommodate hundreds of thousands of transport routes each year.

**Figure 2:**
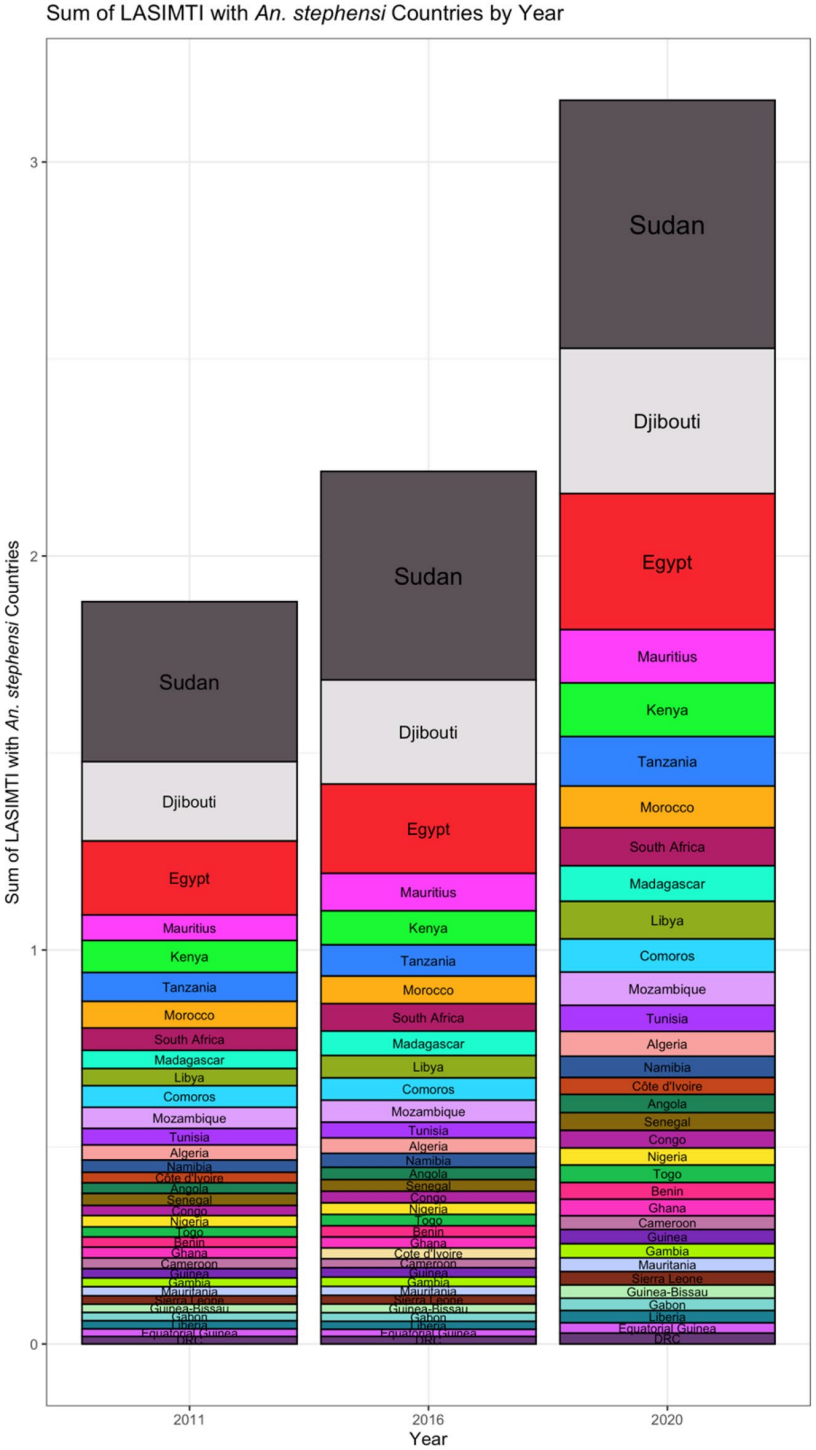
Sum of LASIMTI with *An. stephensi* countries with 2011, 2016, 2020 Trade Data. The sum of each coastal African countries’ Likelihood of *An. stephensi* Introduction through Maritime Trade Index (LASIMTI) with inputs from endemic *An. stephensi* countries sorted in descending order and arranged by year to highlight highly connected countries and overall maritime traffic growth. This graph breaks down the LASIMTI ranking by country by year. Each column is sorted by count LASIMTI sum. This shows that overall maritime trade between endemic *An. stephensi* countries and coastal Africa have increased over time. This also highlights Sudan, Djibouti, Egypt, Mauritius, Kenya, and Tanzania as highly connected countries.

The 2020 version of these data indicate Sudan, Djibouti, Egypt, Kenya, and Mauritius as the top five connected countries when ranked by the sum of LASIMTI (Fig. 1, Supplemental Table 7). Sudan and Djibouti remain the top two connected countries for all the three years examined (Fig. 2). The data utilizing both the HSI and LASIMTI place Sudan, Djibouti, Egypt, Kenya, and Tanzania as the top five countries (Supplemental Table 8).

Since *An. stephensi* populations have been confirmed in Sudan as well in 2019, these data were further examined with Djibouti and Sudan included as potential source populations for *An. stephensi*. With Djibouti and Sudan included as source populations, the top five countries at risk of *An. stephensi* introduction are Egypt, Kenya, Mauritius, Tanzania, and Morocco (Table 2). When the HSI is also included in the ordering, the top five countries are Egypt, Kenya, Tanzania, Morocco, and Libya (Table 3). Full tables can be found in the supplement (Supplemental Tables 9 and 10, respectively).

**Table 2:** Top 10 Countries based on LASIMTI from 2020 UNCTAD data (left).

Coastal African Countries Ranked by *LASIMTI* alone from 2020 UNCTAD Maritime Trade Data	
Rank	African Country
1	Egypt
2	Kenya
3	Mauritius*
4	Tanzania
5	Morocco
6	South Africa
7	Libya
8	Madagascar
9	Mozambique
10	Comoros*

*No HSI data was available for these countries which may contribute to their drop in ranking when HSI and LASIMTI are combined in Table 3.

**Table 3:** Top 10 Countries based on HSI and LASIMTI from 2020 UNCTAD data (right).

Coastal African Countries Ranked by *HSI* and *LASIMTI* from 2020 UNCTAD Maritime Trade Data	
Rank	African Country
1	Egypt
2	Kenya
3	Tanzania
4	Morocco
5	Libya
6	Madagascar
7	Mozambique
8	Angola
9	Senegal
10	Congo

### Intracontinental connectivity network model

**Figure 3:**
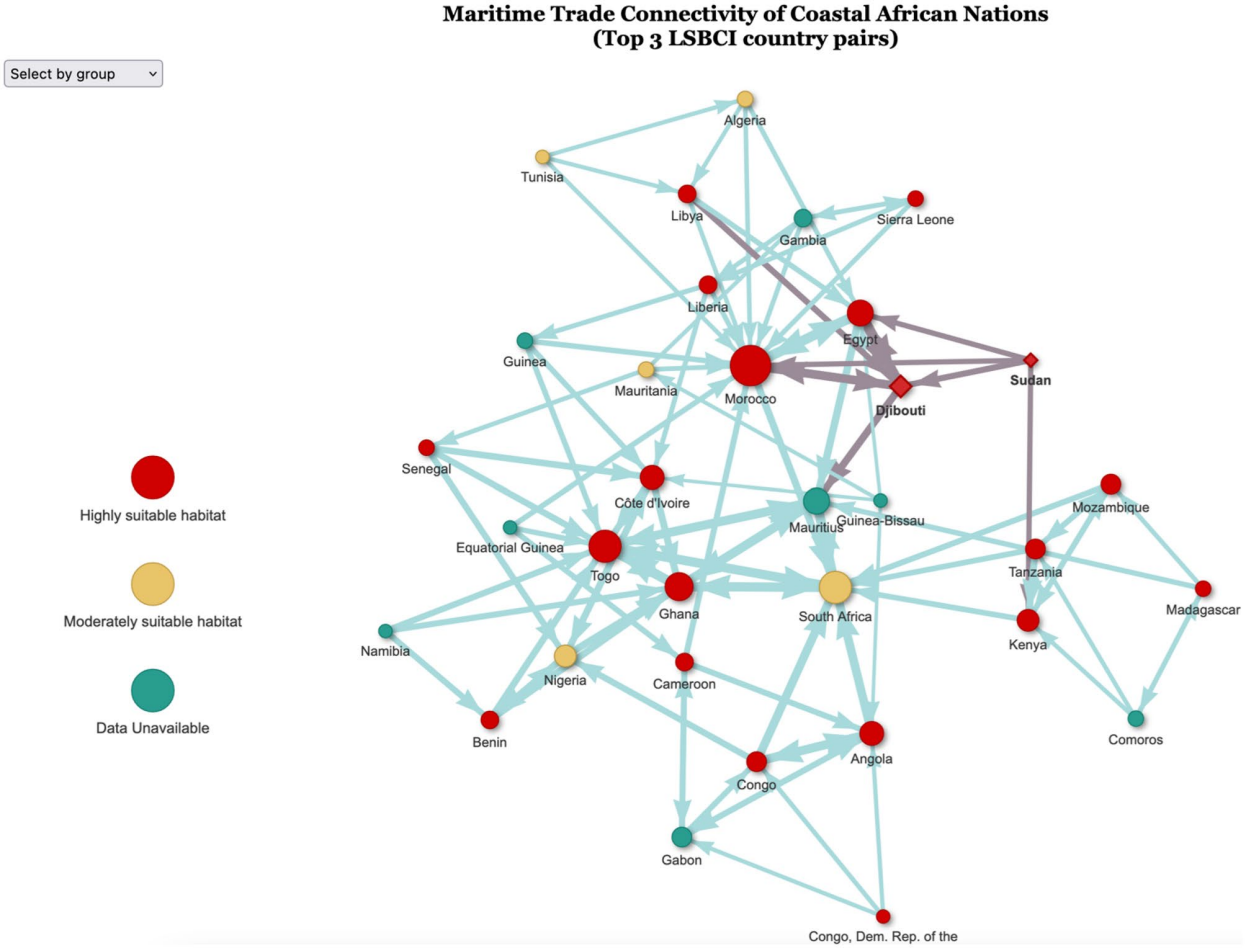
Maritime Trade Network Model with 2020 Trade Data. Directed network model of coastal African nations connected through ranking Liner Shipping Bilateral Connectivity Index (LSBCI) data with Sudan and Djibouti highlighted as having known *An. stephensi* populations. This network model was produced using the 2020 UNCTAD trade index, LSBCI. Each node represents a coastal African country with directed edges pointing towards another node. Darkened edges show connections to countries with confirmed *An. stephensi* as of 2020. A connection indicates an LSBCI ranked as one of the origin node’s highest three LSBCI. The nodes are also weighted by the number of connections directed towards it as shown by the size. The red diamond nodes (Djibouti and Sudan) are countries with known *An. stephensi* populations. (Interactive HTML link found in supplement).

The interactive network model reveals degrees of connectivity within coastal nations on the African continent (Fig. 3). Specifically, it highlights highly connected coastal African countries such as South Africa as well as the Western African nations. Utilizing the PageRank centrality score, South Africa (0.175), Mauritius (0.159), Ghana (0.159), Togo (0.157), and Morocco (0.044) are more highly connected to coastal countries in Africa than others via maritime trade in this network (Supplemental Table 11). Djibouti and Sudan are ranked 7th (0.030) and 32nd (0.0045) respectively. Egypt was highlighted often as being at risk of introduction by the LASIMTI ranking. In the PageRank centrality analysis, Egypt is ranked 6th with a rank value of 0.0353. Other countries that were highlighted are Kenya (11th, 0.0164) and Tanzania (12th, 0.0156).

## Discussion

With human movement and globalization, invasive container breeding vectors responsible for dengue, Zika, chikungunya and now malaria, with *An. stephensi,* are being introduced and establishing populations in new locations. They are bringing with them the threat of increasing or novel cases of vector-borne diseases to new locations where health systems may not be prepared.

*Anopheles stephensi* was first detected on the African continent in Djibouti in 2012 and has since been confirmed in Ethiopia, Somalia, and Sudan. Unlike most malaria vectors, *An. stephensi* is often found in artificial containers and in urban settings. This unique ecology combined with its initial detection in seaports in Djibouti, Somalia, and Sudan has led scientists to believe that the movement of this vector is likely facilitated through maritime trade.

By modeling inter- and intra-continental maritime connectivity in Africa we identified countries with higher likelihood of *An. stephensi* introduction if facilitated through maritime movement and ranked them based on this data. *Anopheles stephensi* was not detected in Africa (Djibouti) until 2012. To determine whether historical maritime data would have identified the first sites of introduction, 2011 maritime data were analyzed to determine whether the sites with confirmed *An. stephensi* would rank highly in connectivity to *An. stephensi* endemic countries. Using 2011 data on maritime connectivity alone, Djibouti and Sudan were identified as the top two countries at risk of *An. stephensi* introduction if it is facilitated by marine cargo shipments. In 2021, these are two of the three African coastal nations where *An. stephensi* is confirmed to be established.

When 2011 maritime data were combined with the HSI for *An. stephensi* establishment, the top five countries remain the same as with maritime data alone: Sudan, Djibouti, Egypt, Kenya and Tanzania, in that order. The maritime data show likelihood of introduction and HSI shows likelihood of establishment. When combined, the analyses show a likelihood of being able to establish and survive once introduced. Interestingly, the results of the combined analyses align with the detection data being reported in the Horn of Africa. The 2011 maritime data reinforces the validity of the model as it points to Sudan and Djibouti, where *An. stephensi* established in the following years. Similarly, the HSI data for Ethiopia has aligned closely with detections of the species to date^15^. Interestingly, around this time of initial detection in Djibouti, Djibouti City port underwent development and organizational change. The government of Djibouti took back administrative control of the port as early as 2012^30^.

**Figure 4:**
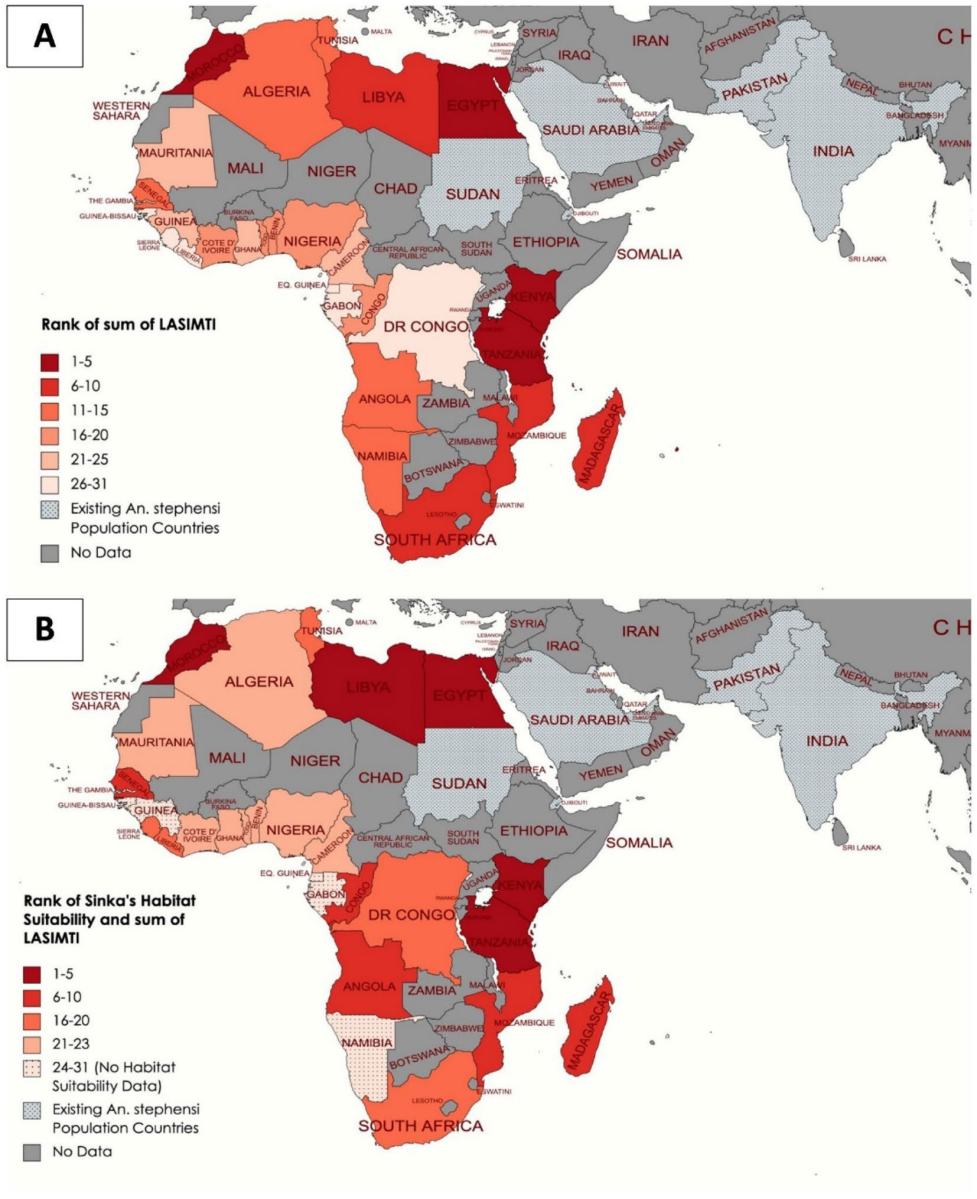
Prioritization Heat Map of African Countries. These 2020 heat maps rank African countries using (**A**) the Likelihood of *An. stephensi* through Maritime Trade Index (LASIMTI) data alone and (**B**) LASIMTI and HSI combined, based on maritime connectivity to countries where *An. stephensi* is endemic. Higher ranking countries which are at greater risk of *An. stephensi* introduction are darker in red color than those that are lower ranking (lighter red). Countries which are shaded grey are inland countries that do not have a coast and therefore no data on maritime movement into ports. Countries which are grey and checkered have established or endemic *An. stephensi* populations and are considered source locations for potential *An. stephensi* introduction in this analysis. Map was generated using MapChart (mapchart.net).

Following this method, maritime trade data from 2020 could point to countries at risk of *An. stephensi* introduction from endemic countries as well as from the coastal African countries with newly introduced populations. Here we provide a prioritization list and heat map of countries for the early detection, rapid response, and targeted surveillance of *An. stephensi* in Africa based on this data and the HSI (Fig. 4). Further invasion of *An. stephensi* on the African continent has the potential to reverse progress made on malaria control in the last century. *Anopheles stephensi* thrives in urban settings and in containers, in contrast to the rural settings and natural habitats where most *Anopheles* spp. are found^20^. The situation in Djibouti may be a harbinger for what is to come if immediate surveillance and control strategies are not initiated^18^.

Maritime data from 2020, with Djibouti and Sudan considered as potential source populations for intracontinental introduction of *An. stephensi,* indicate the top five countries at risk for maritime introduction are Egypt, Kenya, Mauritius, Tanzania, and Morocco, suggesting that targeted larval surveillance in these countries near seaports may provide a better understanding of whether there are maritime introductions. When the data from 2020 data is combined with HSI for *An. stephensi,* the top five countries are instead Egypt, Kenya, Tanzania, Morocco, and Libya. Interestingly, historical reports of *An. stephensi* in Egypt exist; however, following further identification these specimens were determined to be *An. ainshamsi*^31^. With several suitable habitats both along the coast and inland of Egypt, revisiting surveillance efforts there would provide insight into how countries that are highly connected to *An. stephensi* locations through maritime traffic may experience introductions.

Further field validation of this prioritization list is necessary, because it is possible that *An. stephensi* is being introduced through other transportation routes, such as dry ports or airports^32^, or may even be dispersed through wind facilitation^33^. However, countries highlighted here with high levels of connectivity to known *An. stephensi* locations should be considered seriously at risk and surveillance urgently established to determine whether *An. stephensi* introduction has already occurred or to enable early detection. Primary vector surveillance for both *Ae. aegypti* and *An. stephensi* are through larval surveys, and the two mosquitoes are commonly detected in the same breeding habitats. It could therefore be beneficial to coordinate with existing *Aedes* surveillance efforts to be able to simultaneously gather data on medically relevant *Aedes* vectors while seeking to determine whether *An. stephensi* is present. Similarly, in locations with known *An. stephensi* and not well established *Aedes* programs, coordinating surveillance efforts provides an opportunity to conduct malaria and arboviral surveillance by container breeding mosquitoes simultaneously.

Efforts to map pinch points or key points of introduction based on the movement of goods and populations could provide high specificity for targeted surveillance and control efforts. For example, participatory mapping or population mobility data collection methods, such as those used to determine routes of human movement for malaria elimination, may simultaneously provide information on where targeted *An. stephensi* surveillance efforts should focus. Several methods have been proposed in the literature for modeling human movement and one in particular, PopCAB, which is often used for communicable diseases, combined quantitative and qualitative data with geospatial information to identify points of control^34^.

Data on invasive mosquito species has shown that introduction events are rarely a one-time occurrence. Population genetics data on *Aedes* species indicate that reintroductions are very common and can facilitate the movement of genes between geographically distinct populations, raising the potential for introduction of insecticide resistance, thermotolerance, and other phenotypic and even behavioral traits which may be facilitated by gene flow and introgression^35^. Djibouti, Sudan, Somalia, and Ethiopia, countries with established invasive populations of *An. stephensi*, should continue to monitor invasive populations and points of introduction to control and limit further expansion and adaptation of *An. stephensi*. Work by Carter et al. has shown that *An. stephensi* populations in Ethiopia in the north and central regions can be differentiated genetically, potentially indicating that these populations are a result of more than one introduction into Ethiopia from South Asia, further emphasizing the potential role of anthropogenic movement on the introduction of the species^17^.

One major limitation of this work is that Somalia is the third coastal nation where *An. stephensi* has been confirmed; however, marine traffic data were not available for Somalia so it could not be included in this analysis. The potential impact of Somalia on maritime trade is unknown and it should not be excluded as a potential source population. Additionally, this model does not account for the possibility of other countries with *An. stephensi* populations that have not been detected yet. As new data on *An. stephensi* expansion becomes available, more countries will be at higher risk. Other countries with *An. stephensi* populations, such as Iran, Myanmar, and Iraq, constitute lower relative percentages of trade with these countries so were not included in the analysis. However, genetic similarities were noted from *An. stephensi* in Pakistan, so this nation was included^10^.

Due to the nature of maritime traffic, inland countries were also not included in this prioritization ranking. Countries which are inland but share borders with high-risk countries according to the LASTIMI index should also be considered with high priority. For example, the ranking from 2011 highlights Sudan and Djibouti, both which border Ethiopia, and efforts to examine key land transportation routes between bordering nations where humans and goods travel may provide additional insight into the expansion routes of this invasive species.

In Ethiopia, *An. stephensi* was detected in 2016. It has largely been detected along major transportation routes although further data is needed to understand the association between movement and *An. stephensi* introductions and expansion since most sampling sites have also been located along transport routes. Importantly, Ethiopia relies heavily on the ports of Djibouti and Somalia for maritime imports and exports. Surveillance efforts have revealed that the species is also frequently associated with livestock shelters and *An. stephensi* are frequently found with livestock bloodmeals^15^. Interestingly, the original detection of *An. stephensi* was found in a livestock quarantine station in the port of Djibouti. Additionally, livestock constitutes one of the largest exports of maritime trade from this region. For countries with high maritime connectivity to *An. stephensi* locations, surveillance efforts near seaports, in particular those with livestock trade, may be targeted locations for countries without confirmed *An. stephensi* to begin larval surveillance.

As *Ae. aegypti* and *Culex coronator* were detected in tires or *Ae. albopictus* through tire and bamboo (*Dracaena sanderiana)* trade, *An. stephensi* could be carried through maritime trade of a specific good^36–38^. Future examination of the movement of specific goods would be beneficial in interpreting potential *An. stephensi* invasion pathways. Additionally, the various types of vessels used to transport certain cargo such as container, bulk, and livestock ships could affect *An. stephensi* survivability during transit. Sugar and grain are often shipped in bulk or break bulk vessels which store cargo in large unpackaged containers. Container ships transport products stored in containers sized for land transportation via trucks and carry goods such as tires. Livestock vessels are often multilevel, open-air ships which require more hands working on deck and water management^39^.

Using LSBCI index data from 2020, we developed a network to highlight how coastal African nations are connected through maritime trade (Fig. 4). The role of this network analysis is two-fold, (1) it demonstrates an understanding of intracontinental maritime connectivity; and (2) it highlights the top three countries connected via maritime trade through an interactive html model (Supplemental File). For example, if *An. stephensi* is detected and established in a specific coastal African nation such as Djibouti, selecting the Djibouti node reveals the top three locations at risk of introduction from that source country (Djibouti links to Sudan, Egypt and Kenya). This can be used as an actionable prioritization list for surveillance if *An. stephensi* is detected in any given country and highlights major maritime hubs in Africa which could be targeted for surveillance and control. For example, since the development of this model, *An. stephensi* has been detected in Nigeria. Through the use of this interactive model, Ghana, Cote d’Ivoire, and Benin have been identified as countries most connected to Nigeria through maritime trade and therefore surveillance prioritization activities could consider these locations.

The network analysis reveals the significance of South African trade to the rest of the continent. Due to the distance, South Africa did not appear to be high in risk of *An. stephensi* introduction. However, this analysis does reveal that if *An. stephensi* were to enter nearby countries, it could very easily be introduced because of its high centrality. Western African countries such as Ghana, Togo, and Morocco are also heavily connected to other parts of Africa. Interestingly, Mauritius appears to be highly significant to this network of African maritime trade. Based on 2020 maritime data, Mauritius is ranked as the country with the third greatest likelihood of introduction of *An. stephensi* and has the second highest centrality rank value of 0.159. Considering these factors, Mauritius could serve as an important port of call connecting larger ports throughout Africa or other continents. With long standing regular larval surveillance efforts across the island for *Aedes* spp., this island nation is well suited to look for *Anopheles* larvae as part of *Aedes* surveillance efforts for early detection and rapid response to prevent the establishment of *An. stephensi.* If *An. stephensi* were to become established in countries with high centrality ranks, further expansion on the continent could be accelerated drastically. These ports could serve as important watchpoints and indicators of *An. stephensi*’s incursion into Africa. *Anopheles stephensi* is often found in shared habitats with *Aedes* spp. and a great opportunity exists to leverage *Aedes* arboviral surveillance efforts to initiate the search for *An. stephensi,* especially in countries that have high potential of introduction through maritime trade.

## Conclusions

With increases in globalization and volume and frequency of marine cargo traffic connecting countries and continents, information on maritime connectivity can serve as an early warning system for invasive species in general, including those relevant to public health. We show that maritime data prior to the detection of *An. stephensi* in Africa identified Djibouti and Sudan as countries at greatest risk of introduction, and these are locations where invasive *An. stephensi* populations are now established. Using data from 2020 we present a prioritization list of countries at risk of *An. stephensi* introduction through maritime traffic and describe intracontinental maritime connectivity. These data highlight the potential use of maritime trade data for the early detection and rapid response of invasive mosquito vectors, such as *An. stephensi* in Africa, to limit establishment and impact on public health.

The detection of *An. stephensi* in Nigeria, a country with the highest morbidity and mortality due to malaria in Africa and a major urban seaport hub, is concerning^40^. This study emphasizes the importance of leveraging *Aedes* surveillance efforts, conducting surveillance in ports for early detection of the species, and ensuring the predictive risk models, such as our network model here can be iterative any adaptive to include new detections as they arise.

Through integrated vector management, existing *Aedes* programs could be leveraged by providing training for *An. stephensi* identification^41^. Similarly, in locations where *An. stephensi* surveillance is ongoing, the addition of data collection on *Aedes.* spp. should be included for arboviral disease surveillance. These integrated efforts will strengthen local, regional, and national entomological surveillance systems for vector borne diseases.

## Supplementary Information

Supplementary Information 1.

Supplementary Information 2.

## Data Availability

All data generated are included in this manuscript and supplementary files.

## Abbreviations

LSBCI: Liner Shipping Bilateral Connectivity Index
LASIMTI: Likelihood of *Anopheles stephensi* Introduction Through Maritime Trade Index
